# CD19^+^ B-Cells, a New Biomarker of Mortality in Hemodialysis Patients

**DOI:** 10.3389/fimmu.2018.01221

**Published:** 2018-06-15

**Authors:** María Molina, Luis M. Allende, Luis E. Ramos, Eduardo Gutiérrez, Daniel E. Pleguezuelo, Eduardo R. Hernández, Francisco Ríos, Cristina Fernández, Manuel Praga, Enrique Morales

**Affiliations:** ^1^Department of Nephrology, Hospital Universitario 12 de Octubre and Research Institute i+12, Madrid, Spain; ^2^Department of Immunology, Hospital Universitario 12 de Octubre and Research Institute i+12, Madrid, Spain; ^3^San Luciano Hemodialysis Unit, Fresenius Medical Care, Madrid, Spain; ^4^Department of Epidemiology and Preventive Medicine, Hospital Universitario Clínico San Carlos, Universidad Complutense de Madrid, Madrid, Spain; ^5^Department of Medicine, Universidad Complutense de Madrid, Madrid, Spain

**Keywords:** B-cells, lymphocytes, hemodialysis, mortality, cardiovascular disease

## Abstract

**Background and objectives:**

Mortality of patients on hemodialysis (HD) remains very high despite recent improvements in HD techniques. Cardiovascular (CV) complications and infections are the main causes of death. Some studies suggest that disturbances in the immune system could play a role in this disproportionate mortality, through the links of immunity with inflammation and propensity to infections. However, few studies have addressed the role of lymphocyte populations and the global and CV mortality of HD patients.

**Aim:**

To analyze the relationship of peripheral blood lymphocyte populations (PBLP) and all-cause and CV mortality of HD patients.

**Design, setting, participants, and measurements:**

We design a prospective observational single center study in a cohort of HD prevalent patients. PBLP were analyzed at baseline and after 1 year and patients were followed for a 5-year period. Main outcomes were all-cause and CV mortality.

**Results:**

One hundred and four patients (51% male, mean age 64.8 ± 15 years) were included. Follow-up was 18 (7–47) months. Fifty-five patients (52.8%) died, main causes of death being CVD (40%) and infections (29.1%). Low total lymphocyte counts were found in 47 patients (45.2%), and the most frequency lymphopenias were CD19^+^ B-cell (57.7%), CD3^+^ (40.4%), and CD4^+^ (36.5%). After 1 year, all determinations were lower except CD56^+^CD16^+^CD3^−^ natural killer. Patient survival was significantly lower in patients with a CD19^+^ B-cell count < 100 cells/μL at baseline as compared to patients with CD19^+^ B-cell ≥ 100 cells/μL counts at the end of follow-up (16.5 vs 54%, *p* = 0.003). By multivariable analysis, age, history of CV disease, Charlson index, a KT/V < 1.2, and a CD19^+^ B-cell count < 100 cells/μL at baseline and after 1-year were factors associated with of all-cause mortality. A CD19^+^ B-cell count < 100 cells/μL at baseline was associated with CV mortality.

**Conclusion:**

CD19^+^ B-cell lymphopenia is very common among HD patients, and it could be an independent predictor of all-cause and CV mortality. More studies are needed to confirm these findings.

## Introduction

Despite recent technical advances, the mortality of patients undergoing hemodialysis (HD) continues to be very high. In Spain, annual mortality is 15%, ranging from 3% in patients younger than 45 years to 24% in patients older than 75 years (1–3). Our country ranks middle in the HD worldwide mortality that oscillates between 10% in the ANZDATA registry (4), 16% in the European countries (5), and 20% in USA (6). The main causes of HD mortality in Spain are infections (more than 20%), followed by cardiovascular (CV) disease (18%) and unknown causes (13%) (1).

In the last decades, many studies have demonstrated abnormalities in different components and function of the immune system in HD patients (7–13). Total lymphopenia has been shown to be a predictor of all-cause mortality in HD (14–16). HD patients show an abnormal immune response, with a reduced response to certain pathogens and vaccines, increased susceptibility to infections caused by intracellular pathogens, increased tolerance to cutaneous grafts, and a higher incidence of tumors as compared with the general population (17–24).

Over the last few years, the relationship between the immune system and CV disease has gained a great prominence, generating an intense research about the influence of immune abnormalities on the pathogenesis of CV disease and the possible discovery of new therapeutic approaches (25). This is an area of special interest in patients with chronic kidney disease (CKD), since many of the classic risk factors for CV disease in the general population do not have the same influence in CKD patients (15, 26). The lymphocytes subpopulations are divided between CD3^+^ T-cell, CD19^+^ B-cell, and CD56^+^CD16^+^CD3^−^ natural killer (NK) cells. There are two groups of T-cell lymphocyte, CD8^+^ and CD4^+^. Several cellular subtypes and immunoglobulins have a proatherosclerotic influence, whereas others have a protective role (25, 27–31). The uremic environment produces an inflammatory condition that promotes early atheromatous lesions, similar to autoimmune diseases such as rheumatoid arthritis and systemic lupus erythematosus (32–35). Some studies have analyzed the role of different CD3^+^ T-cell lymphocytes subtypes and the cytokines they synthetize in the appearance and progression of CV disease in HD patients (28, 29, 36, 37). However, no information about the participation and influence of B-cells on CV disease of patients undergoing HD has been reported as far as we know. The aim of this study was to analyze the relationship between peripheral blood lymphocyte populations (PBLP) and the mortality of HD patients.

## Patients and Methods

### Patients

We designed a prospective, single-center observational study. All the patients undergoing HD in the Hospital Universitario 12 de Octubre and San Luciano Center (an HD facility operated by Fresenius Medical Care and linked to Hospital Universitario 12 de Octubre) were included in the study. The only exclusion criteria was an onset of chronic HD shorter than 30 days before the study. All procedures were performed according to good clinical practice guidelines and all patients gave their written informed consent for study participation (38).

Baseline was set at the time of extraction of blood samples for the determination of lymphocyte populations, immunoglobulins, and complement factors (see below). Clinical and analytical variables selected for the study were recorded at baseline. In patients needing hospitalization and in those presenting acute intercurrent illnesses, baseline studies were delayed until 1 month at least after recovery of the acute event. Baseline determinations were performed between April 2011 and April 2012. After inclusion and baseline determinations, patients were followed for a maximum of 5 years. Patients transferred to other HD facilities and those who received a kidney graft were censored.

### Variables

Demographic and clinical variables included age, gender, CKD etiology, previous renal transplantation (RT), months on HD, comorbidities (arterial hypertension, diabetes mellitus, heart failure, stroke, tumors), and the Charlson comorbidity index (39). Previous immunosuppression, treatment with erythropoietin stimulating agents or vitamin D, type of vascular access (arteriovenous fistula or central venous catheter), a positive serology for hepatitis C virus, and whether the patient was on the waiting list for a kidney transplant were also recorded.

The following analytical parameters were collected at baseline: hemoglobin, total proteins, albumin, calcium, phosphorus, C-reactive protein, parathormone, and 25-hydroxy-vitamin D.

Prescription of HD sessions was based on a regimen of 3 sessions per week (4 h each). The characteristics of HD sessions were recorded, including duration, type of HD (conventional or hemodiafiltration) and Kt/V.

### PBLP, Immunoglobulins, and Complement Factors

Peripheral blood lymphocyte populations (total lymphocytes and the following lymphocyte populations: CD3^+^ T-cell, CD4^+^ T-cell, CD8^+^ T-cell, CD19^+^ B-cells, and CD56^+^CD16^+^CD3^−^ NK), immunoglobulins (IgG, IgA, and IgM), and complement factors (C3 and C4) were extracted at the onset of a HD session. Blood samples were collected and analyzed within 18 h at the Department of Immunology. Whole blood (50 µL) was stained with 20 µL of BD Multitest 6-color TBNK reagent in Trucount tubes for 15 min. Red blood cells were lysed using fluorescence activated cell sorting lysing solution. Determination of PBLP was performed with a FACSCanto II flow cytometer, and data analyzed by FACSCanto clinical software (BD Biosciences, San Jose, CA, USA) (40). Values were compared with the range of normality established by our laboratory using blood samples from healthy controls.

### Outcomes

The primary outcome measure was all-cause mortality. Secondary outcomes were CV death (defined as death related to myocardial infarction, heart failure, and cerebrovascular or peripheral vascular disease).

### Statistical Analyses

Continuous variables were expressed as mean ± standard deviation (SD) or the median [interquartile range (IQR)] as appropriate, and they were analyzed with Student’s *t*-test or *U* Mann–Whitney test, as proceeded. Qualitative variables were showed as absolute and relative frequencies and they were compared with the chi-square test. Patient survival was drawn by the Kaplan–Meier method and different groups were compared with the Log Rank test. Patients were censured from analysis if they received a RT, transferred to peritoneal dialysis or other facilities. When we analyzed factors associated with CV death, we censured for other causes of death. We performed a multivariate analysis using Cox regression model (backward conditional selection). We defined an explicative model. Interaction between variables was explored. We included in multivariate analysis all variable with *p* value < 0.10 in univariate analysis and clinical relevance. Results shown as hazard ratios (HR) and the corresponding 95% confidence intervals (CI). We consider as statistical significance a *p* value < 0.05. The statistical analysis was performed with the program SPSS v. 20.0 for Mac (SPSS, Chicago, IL, USA).

## Results

### Baseline Characteristics

**Figure 1:**
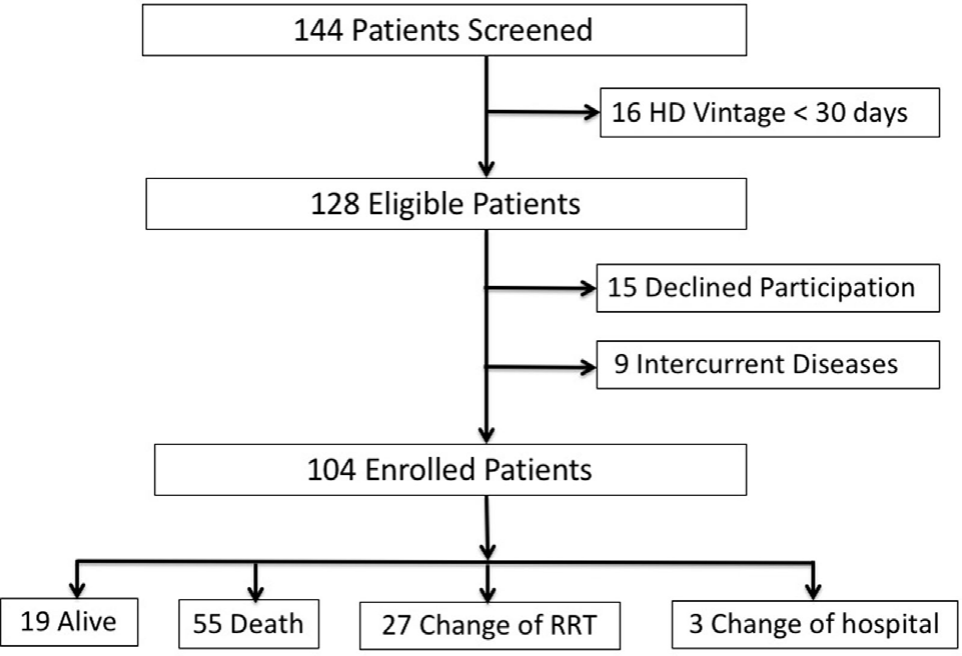
Flow-chart. Abbreviations: HD, hemodialysis; RRT, renal replacement therapy.

One hundred and forty-four patients were selected for the study. Forty patients were excluded because a HD vintage < 30 days (16 patients), refusal to sign informed consent (15 patients), and non-resolved intercurrent diseases (9 patients). One hundred and four patients were finally enrolled in the study (Figure 1).

The main baseline characteristics are shown in Table 1. Mean age was 64.8 ± 15 years, and 35 patients (33.7%) were 75 years or older. Mean Charlson index was 6.5 ± 2.8 (median 6.5 IQR 5–8). Twenty-eight patients (26.9%) had history of tumors (renal in 6, skin in 4, bladder in 3, breast in 3, colon in 3, prostate in 2, multiple myeloma in 2, tongue in 1, thyroid gland in 1, neuroendocrine tumor in 1, gastrointestinal stromal tumor in 1, and of unknown origin in 1). Forty-six patients (44.2%) had received immunosuppression because a previous RT in 32, non-RT in 3, glomerulonephritis in 5, autoimmune diseases in 4, and other different causes in 2. Patients had started HD 34 (IQR 8–83) months before the study. More than half of the patients (53.8%) had a central venous catheter as vascular access for HD at baseline.

**Table 1:** Baseline characteristics of the study group.

Variables	Patients (104)
Gender (male)^a^	53 (51)
Age at baseline, years^b^	64.8 ± 15
**CKD etiology^a^**	
Glomerulonephritis	22 (21.2)
Hypertension/vascular	22 (21.2)
Diabetes	21 (20.2)
Tubulointersticial nephritis	8 (7.7)
ADPKD	5 (4.8)
Unknown	3 (2.9)
Hypertension^a^	79 (76)
Diabetes^a^	31 (29.8)
Dyslipidemia^a^	54 (51.9)
History of cardiovascular disease^a^	62 (59.6)
History of peripheral vascular disease^a^	26 (25)
History of cancer^a^	28 (26.9)
History of kidney transplantation^a^	32 (30.8)
Charlson index^b^	6.5 ± 2.8
Hepatitis C^a^	23 (22.1)

Central venous catheter^a^	56 (53.8)
Hemodiafiltration^a^	46 (44.2)
Time on hemodialysis, months^c^	34 (8–83)
Kt/V^b^	1.6 ± 0.3

Hemoglobin, g/dL^b^	11.8 ± 1.3
Albumin, g/dL^b^	3.9 ± 0.4
Calcium, mg/dL^b^	9.1 ± 0.6
Phosphorus, mg/dL^b^	4.8 ± 1.6
PTH, pg/mL^c^	226 (149–440)
C Reactive Protein, mg/dL^c^	0.8 (0.4–1.8)

*^a^*n* (%)*.

*^b^Mean ± SD*.

*^c^Median (interquartile range)*.

*ADPKD, autosomal dominant polycystic kidney disease; PTH, parathormona; CKD, chronic kidney disease*.

### PBLP, Immunoglobulins, and Complement Factors

**Figure 2:**
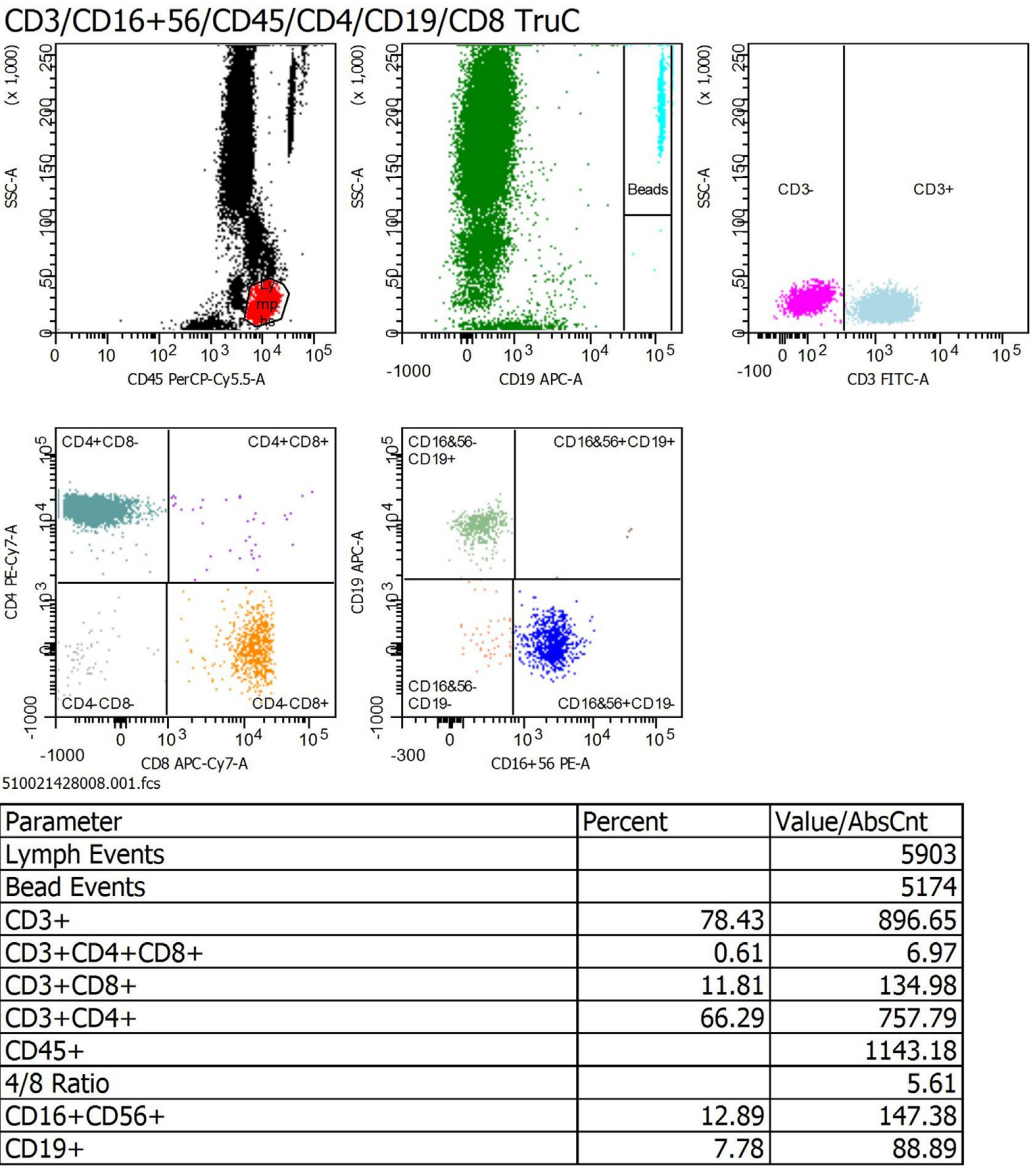
Representative schema of a peripheral blood lymphocyte populations FACS profile in a hemodialysis patient.

Figure 2 shown a representative schema of a PBLP determination in one HD patient. The values of PBLP are shown in Table 2. Low total lymphocytes counts were found in 47 patients (45.2%). CD3^+^ T-cell, CD4^+^ T-cell, and CD8^+^ T-cell counts were low in 40.4, 36.5, and 10.6%, respectively. Decreased counts of CD19^+^ B-cells and CD56^+^CD16^+^CD3^−^ NK lymphocytes were found in 57.7 and 8.7% of the patients, respectively (see Table 2).

**Table 2:** Lymphocyte populations, immunoglobulins, and complement results.

Variable	Patients (*n* = 104)	Normal lab range	Number of patients (%) with values below normal ranges
Total lymphocytes (cells/μL)^b^	1,500 ± 889	1,200–3,000	47 (45.2)^a^
CD3^+^ T-cells (cells/μL)^c^	957 (667–1,461)	850–2,250	42 (40.4)^a^
CD4^+^ T-cells (cells/μL)^b^	637.2 ± 379	500–1,450	38 (36.5)^a^
CD8^+^ T-cells (cells/μL)^c^	354 (226–596)	160–950	11 (10.6)^a^
CD4^+^/CD8^+^ ratio^b^	1.6 ± 0.8	1–3	25 (24)^a^
CD19^+^ B-cells (cells/μL)^c^	89 (49–140)	100–500	60 (57.7)^a^
CD56^+^CD16^+^CD3^−^ NK (cells/μL)^c^	174 (103–285)	60–450	9 (8.7)^a^
IgG (mg/dL)^b^	1,161.4 ± 466	700–1,600	14 (13.5)^a^
IgA (mg/dL)^b^	287.4 ± 139	70–400	0 (0)^a^
IgM (mg/dL)^c^	77 (52–129)	40–230	13 (12.5)^a^
C3 (mg/dL)^b^	90.4 ± 21.1	83–171	37 (35.6)^a^
C4 (mg/dL)^b^	22.4 ± 5.5	14–38	4 (3.8)^a^

*^a^*n* (%)*.

*^b^Mean ± SD*.

*^c^Median (interquartile range)*.

*NK, natural killers*.

IgG and IgM hypogammaglobulinemia was detected at baseline in 13.5 and 12.5% of the patients, respectively. Decreased values of serum complement factor C3 and C4 at baseline were found in 35.6 and 3.8% of patients, respectively.

A second determination of immunoglobulin, complement, and PBLP was performed 12 months after baseline in 46 patients. There were no significant changes in serum immunoglobulins and complement values. As compared to baseline values, there was a significant decrease in total lymphocyte count [from 1,161 (854–1,842) to 1,029 (735–1,566) cells/μL, *p* = 0.002], CD3^+^ T-cells [from 845 (539–1,484) to 791 (480–1,182) cells/μL, *p* = 0.004], CD4^+^ T-cells [from 553 (279–715) to 440 (287–700) cells/μL, *p* = 0.009], and CD8^+^ T-cells [from 348 (193–581) to 286 (171–434) cells/μL, *p* = 0.01]. CD19^+^ B-cells also showed a significant decrease, from 81 (34–121) to 59 (29–115) cells/μL (*p* = 0.01), whereas CD56^+^CD16^+^CD3^−^ NK lymphocytes did not show significant changes.

### Outcomes

Median follow-up was 18 (7–47) months. As shown in Figure 1, one patient was transferred to peritoneal dialysis (PD), 26 patients received a RT, and 3 patients were transferred to other centers. There were 55 deaths at the end of follow-up, 22 (40%) of them attributed a CV causes and 16 (29%) to infections. Other causes of death were tumors (7 patients, 12.7%), sudden death (3 patients, 5.4%), and others (7 patients, 12.7%).

We compared PBLP between patients who were alive vs death. Patients who died had lower recount of CD19^+^ B-cells than alive patients (Table 3).

**Table 3:** Lymphocyte populations, immunoglobulins, and complement results and mortality.

Variable	Alive (49)	Deaths (55)	*p*-Value
Total lymphocytes (cells/μL)^b^	1,299 (905–1,862)	1,132 (855–1,560)	0.36
CD3^+^ T-cells (cells/μL)^b^	1,005 (683–1,481)	839 (568–1,256)	0.24
CD4^+^ T-cells (cells/μL)^b^	641 (341–808)	558 (317–651)	0.25
CD8^+^ T-cells (cells/μL)^b^	366 (237–598)	279 (179–455)	0.20
CD4^+^/CD8^+^ ratio^a^	1.6 ± 0.8	1.8 ± 0.9	0.43
CD19^+^ B-cells (cells/μL)^b^	99 (53–158)	67 (34–81)	0.02
CD56^+^CD16^+^CD3^−^ NK (cells/μL)^b^	173 (99–284)	199 (109–352)	0.54
IgG (mg/dL)^a^	1,153 ± 492	1,183 ± 382	0.81
IgA (mg/dL)^a^	279 ± 128	313 ± 179	0.34
IgM (mg/dL)^b^	77 (53–123)	77 (51–136)	0.88
C3 (mg/dL)^a^	91 ± 22	91 ± 18	0.95
C4 (mg/dL)^a^	22.7 ± 5	20 ± 6	0.06

*^a^Mean ± SD*.

*^b^Median (interquartile range)*.

*Ig, immunoglobulin; NK, natural killers*.

As shown in Table 4, age, history of CV disease, Charlson index, a KT/V < 1.2, and a serum albumin < 3.5 mg/dL were variables significantly associated with a higher all-cause mortality in the univariate analysis. On the contrary, being included in the waiting list for RT was a protective factor for survival. There was a non-significant trend to higher all-cause mortality in patients with central vascular catheter as vascular access for HD and in those with pulmonary hypertension, whereas hemodiafiltration and treatment with paricalcitol showed a non-significant protective trend (Table 4). Regarding PBLP, a CD19^+^ B-cells count below 100 cells/μL at baseline and at 1 year, were significantly associated with all-cause mortality. There was a non-significant trend to higher mortality in patients with low total lymphocyte and CD8^+^ T cells counts as well as CD56^+^CD16^+^CD3^−^ NK cells at baseline.

**Table 4:** Univariate and multivariate analysis for all-cause mortality.

Variable	Alive (49)	Deaths (55)	*p*-Value	Univariate			Multivariate		
				HR	95% CI	*p*-Value	HR	95% CI	*p*-Value
Age^b^	58 ± 14.8	71 ± 13	<0.001	1.05	1.02–1.07	<0.01	1.03	1.005–1.05	0.01
Waiting list for KT^a^	27 (55)	6 (11)	<0.001	0.3	0.1–0.7	0.008	–	–	–
CV disease^a^	21 (43)	41 (74.5)	0.001	2.7	1.5–5	0.001	2	1.08–3.9	0.02
Pulmonary hypertension^a^	7 (14)	16 (29)	0.02	1.7	0.98–3.1	0.06	–	–	–
Charlson index^b^	5.4 ± 2.4	7.5 ± 2.6	<0.01	1.14	1.05–1.2	0.002	1.1	1.004–1.2	0.04
KT/V < 1.2^a^	5 (10)	11 (20)	0.18	2.8	1.4–5.5	0.004	2.3	1.1–4.9	0.02
Central venous catheter^a^	25 (51)	31 (56)	0.32	1.6	0.9–2.7	0.09	–	–	–
Paricalcitol treatment^a^	29 (59)	26 (47)	0.15	0.6	0.3–1	0.05	–	–	–
Hemodiafiltration^a^	28 (57)	18 (33)	0.007	0.6	0.3–1	0.05	–	–	–
Albumin < 3.5 mg/dL^a^	3 (6)	13 (24)	0.019	2.8	1.5–5.3	0.002	1.9	1–3.8	0.05
C reactive protein, mg/dL^c^	0.7 (0.3–1.6)	0.8 (0.5–2.2)	0.11	1.05	0.99–1.1	0.14	–	–	–
Total lymphocytes (cells/μL)^c^	1,303 (1,013–2.200)	1,200 (827–1,700)	0.15	1	0.99–1	0.08	–	–	–
CD8^+^ T-cells < 160 células/μL^a^	3 (6)	8 (15)	0.13	1.8	0.9–3.9	0.11	–	–	–
CD19^+^ B-cells < 100 cells/μL^a^	20 (41)	40 (73)	0.001	2.5	1.3–4.6	0.004	2	1.05–3.8	0.03
CD19^+^ B-cells < 100 cells/μL after 1 year^a^	12 (50)	21 (88)	0.01	5	1.5–17	0.009	3.8	1.005–14	0.04
CD56^+^CD16^+^CD3^−^ NK cells < 60 cells/μL^a^	2 (4)	7 (13)	0.11	2.1	0.9–4.8	0.06	2.4	1.06–5.5	0.03
CD56^+^CD16^+^CD3^−^ NK cells < 60 cells/μL after 1 year^a^	1 (4)	2 (8)	–	2.1	0.5–9.1	0.31	–	–	–

*^a^*n* (%)*.

*^b^Mean ± SD*.

*^c^Median (interquartile range)*.

*CV, cardiovascular; KT, kidney transplantion; NK, natural killers*.

By multivariable analysis (Table 4), age as continuous variable (HR 1.03, 95% CI: 1.005–1.05, *p* = 0.01), history of CV disease (HR 2, 95% CI, 1.08–3.9, *p* = 0.02), Charlson index as continuous variable (HR 1.1, 95% CI: 1.004–1.2, *p* = 0.04), a KT/V < 1.2 (HR 2.3, 95% CI: 1.1–4.9, *p* = 0.02), a CD19^+^ B-cells count < 100 cells/μL at baseline (HR 2, 95% CI: 1.05–3.8, *p* = 0.03) and at 12 months (HR 3.8, 95% CI: 1.005–14, *p* = 0.04), and a CD56^+^CD16^+^CD3^−^ NK cells count < 60 cells/μL at baseline (HR 2.4, 95% CI: 1.06–5.5, *p* = 0.03) were factors significantly associated with a higher risk of all-cause mortality. A serum albumin < 3.5 g/dL was almost significantly associated with an increased risk of mortality (HR 1.9, 95% CI 1–3.8, *p* = 0.05), whereas CD56^+^CD16^+^CD3^−^ NK lymphopenia at 12 months did not show significant association with mortality.

**Figure 3:**
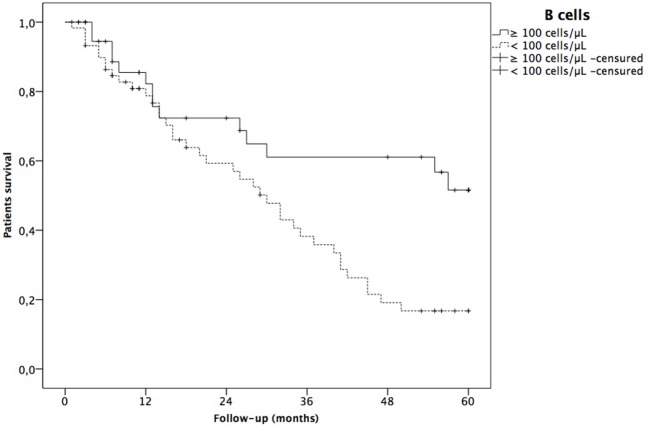
Kaplan–Meier survival curves for number of CD19^+^ B-cells (< or ≥100 cells/μL) and global patient survival were analyzed with a log-rank test.

Patients who died had lower recount of CD19^+^ B-cells than alive patients (Table 3). As shown in Figure 3, survival was significantly lower in patients with a CD19^+^ B-cells count < 100 cells/μL at baseline (79.1, 37.4, and 16.4% at 12, 36, and 60 months of follow-up, respectively) as compared to patients with a CD19^+^ B-cell count ≥ 100 cells/μL at baseline (82.7, 62.4, and 54% at 12, 36, and 60 months of follow-up, respectively; Log Rank 9, *p* = 0.003).

Regarding secondary outcomes, we compared PBLP between patients who were alive vs CV death (data not shown). A CD19^+^ B cell count < 100 cells/μL at baseline increased the risk to CV death (HR 4.1, IC 95%: 1.2–14.6, *p* = 0.02), together with ischemic heart disease (HR 3.5, IC 95%: 1.4–9, *p* = 0.008), Charlson index measured as continuous variable (HR 1.16, IC 95%: 1.02–1.3, *p* = 0.02), and age (HR 1.04, IC 95%: 1.006–1.1, *p* = 0.02) (Table 5).

**Table 5:** Multivariate analysis for cardiovascular deaths.

Variables	HR	95% CI	*p*-Value
CD19^+^ B-cells < 100 cells/μL	4.1	1.18–14.6	0.02
Ischemic heart disease	3.5	1.4–9	0.008
Charlson index	1.16	1.02–1.3	0.02
Age	1.04	1.006–1.1	0.02

## Discussion

The high mortality of patients undergoing HD, mainly related to CV complications, is a global concern (1, 2, 4–6). Previous studies have shown that a low total lymphocyte count is a predictor of global death in HD patients (14–16), related to infections and the appearance of tumors (17–24). However, no studies have analyzed the link of the different lymphocyte subpopulations with the mortality of these patients.

We conducted a prospective study in a cohort of prevalent HD patients in whom an analysis of lymphocyte subpopulations was performed at baseline and after 1 year of follow-up. The patients were followed for a median of 18 (7–47) months, and during this period of follow-up, all deaths were carefully recorded, specifying their cause. A trend toward higher mortality risk associated with total lymphopenia was observed, although it did not reach statistical significance. Although, we found that the patients with low CD19^+^ B-cells count could have higher all-cause and CV mortality in this cohort of patients. This finding could have a remarkable clinical significance, considering that the prevalence of B-cell lymphopenia among HD patients is very high (3, 9–13, 41, 42): 57.7% of our patients had a CD19^+^ B cell count < 100 cells/μL, and this selective B-cell lymphopenia worsened after 12 months of follow-up. This association persists when we analyze CD19^+^ B-cells count after 12 months.

B-cell lymphopenia is common among HD patients and is due to an increased apoptosis mediated by a decreased Bcl-2 expression and a resistance to IL-7 and B-cell-activating factor of the TNF family, both necessary for the differentiation and survival of B-cells (12, 42). Patients on HD have been reported to have a decrease in both B1 (atheroprotective) and B2 (proatherogenic) lymphocytes, although the reduction is greater in B1 lymphocytes (12). A study performed in children undergoing HD showed a low count of B1 cells in comparison with healthy children (10).

The main cause of death in our cohort was CV disease. The implications of the immune system and lymphocyte subpopulations in the development of atherosclerosis and the occurrence of CV events is a very complex issue, although in recent years some of their pathogenic mechanisms have been partially clarified. While the first step in the development of atherosclerosis is the infiltration of low-density lipoprotein (LDL) in the arterial intima, cumulative evidence implicates immune cells in this process. Some lymphocyte types, such as CD4^+^ T-cells, CD56^+^CD16^+^CD3^−^ NK lymphocytes, CD8^+^ T-cells, and B2 cells, have a proatherogenic influence; others, like regulatory T-cells (Tregs), B1a cells, Innate response activator (IRA), and regulatory B-cells (Bregs), play a protective role for the CV system. On the other hand, IgG and IgE immunoglobulins are proatherogenic, while IgM has a protective influence (27–31, 43, 44). CD8^+^ T and CD56^+^CD16^+^CD3^−^ NK cells are present in the earlier phases of the atherosclerotic process, but CD4^+^ T-cells have a more prominent role in the atherosclerotic damage. CD4^+^ T-cells are divided in four groups: T helper (T_H_) 1, T_H_2, T_H_17, and Tregs cells. T_H_1 lymphocytes are frequently found in atherosclerotic plaques, where they recruit and activate macrophages, promote foam cell formation and induce a direct vascular damage through the secretion of interferon-γ (25, 27–30). An increase in the number of T_H_17 cells and a decrease in Tregs has been reported in uremic patients, and this imbalance could collaborate in the appearance of CV complications (36, 37).

Our study suggests that the patients with lower recount of CD19^+^ B-cells have more risk to all-cause and CV mortality. The explanation of this association exceeds the propose of our study. Furthermore, the role of B-cells in the development of CV complications in CKD patients is largely unknown. B-cells are classified into different subtypes: B1 (B1a and B1b) cells that secrete IgM and IgA, and B2 cells that produce IgG. Some studies in murine models suggest that B2 lymphocytes collaborate in atherosclerotic lesions through IgG-mediated LDL oxidation, although these findings are controversial. In addition, B2 lymphocytes polarize and activate T_H_1 response. B1a, IRA, and Bregs in mice would have a protective CV influence because the humoral response could bind to oxidative-LDL and prevent the formation of foam cells. Unfortunately, the translation of the murine model to human is limited. The are some evidence of the role of B-cell in CDV in humans: splenectomized patients has increased risk of death owing a myocardial infarction, some genes related with B-cells have been related with CDV events and IgM-mediated LDL have an inverse association with atherosclerosis (25, 30, 31, 43–45).

Our study has important limitations. It is an observational study and only describe the evolution of HD patients with low number of CD19^+^ B-cells and suggests a possible association with global and CV deaths. It is necessary to perform other studies. Our institution had initiated two studies to confirm these findings. The number of included patients was relatively small and patients on PD or earlier stages of CKD were not included. We only did two determinations of PBPL in a cohort of prevalent HD patients, but the kinetics of the lymphocytes may need more measurements. Some factors could influence in the recount of lymphocytes, we did a lineal regression analysis and couldn’t find any relationship but it could exist. The proportion of the different subpopulations of B lymphocytes was not studied. We defined CV death as the death related to myocardial infarction, heart failure, cerebrovascular, or peripheral vascular disease. Some of these events could be initiated by HD process itself, as arrhythmia or hypotension and not directly related with immune system. The optimal analysis to CV vs other causes of death should be a competitive risk assessment, and it wasn’t done. Although the study has several limitations, it has too some advantages. The study is the first that prospectively analyzes the influence of lymphocyte subpopulations on the mortality of a cohort of prevalent HD patients, without exclusions for comorbidities or previous treatments, and who were carefully followed by an extended period of time. Although HD patients have several concurrent factors that increase death, we performed a multivariable analysis and corrected for other variables. This is the first step to develop new lines of investigation about different lymphopenias and mortality of HD and other stages of CKD.

In conclusion, HD patients exhibit a high incidence of lymphopenia affecting different lymphocyte populations, especially CD19^+^ B-cell. Our data suggest that B-cell lymphopenia could be a predictor of all-cause and CV mortality. Further research is needed to uncover the mechanisms of this association and to evaluate its possible therapeutic implications.

## Ethics Statement

All procedures were performed according to good clinical practice guidelines and all patients gave their written informed consent for study participation.

## Author Contributions

MM and EM contributed conception and design the study; MM, LA, LR, EG, DP, FR, and EM performed the research; MM, EM, and CF performed the statistical analysis; MM wrote the manuscript; LA, EH, CF, MP, and EM revised and completed the final draft of the article. All authors approved the submitted version.

## Conflict of Interest Statement

The authors declare that the research was conducted in the absence of any commercial or financial relationships that could be construed as a potential conflict of interest.

## Abbreviations

CI, confidence intervals; CKD, chronic kidney disease; CV, cardiovascular; HD, hemodialysis; Ig, immunoglobulin; IQR, interquartile range; NK, natural killer; PBLP, peripheral blood lymphocyte populations; SD, standard deviation; PD, peritoneal dialysis; RT, renal transplantation; LDL, low-density lipoprotein; Tregs, regulatory T-cells; Bregs, regulatory B-cells; IRA, innate response activator; TH, T helper.
